# Monoterpene antifungal activities: evaluating geraniol, citronellal, and linalool on *Candida* biofilm, host inflammatory responses, and structure–activity relationships

**DOI:** 10.3389/fphar.2024.1394053

**Published:** 2024-07-19

**Authors:** Priscilla Guimarães Silva Vasconcelos, Kyu Min Lee, Gabriel Flores Abuna, Edja Maria Melo Brito Costa, Ramiro Mendonça Murata

**Affiliations:** ^1^ Department of Dentistry, Postgraduate Program in Dentistry, State University of Paraiba, Campina Grande, Paraíba, Brazil; ^2^ Department of Foundational Sciences, School of Dental Medicine, East Carolina University, Greenville, NC, United States

**Keywords:** structure–activity relationship, antifungal agents, anti-inflammatory agents, oral candidiasis, denture stomatitis

## Abstract

**Introduction:** Despite the rising concern with fungal resistance, a myriad of molecules has yet to be explored. Geraniol, linalool, and citronellal are monoterpenes with the same molecular formula (C_10_H_18_O), however, neither the effect of these compounds on inflammatory axis induced by *Candida* spp. nor the antibiofilm Structure-Activity Relationship (SAR) have been well-investigated. Herein we analyzed geraniol, linalool and citronellal antifungal activity, cytotoxicity, and distinctive antibiofilm SAR, also the influence of geraniol on *Candida* spp induced dysregulated inflammatory axis, and *in vivo* toxicity.

**Methods:** Minimal inhibitory (MIC) and fungicidal (MFC) concentrations against *Candida* spp were defined, followed by antibiofilm activity (CFU–colony forming unit/mL/g of dry weight). Cytotoxic activity was assessed using human monocytes (THP-1) and oral squamous cell (TR146). Geraniol was selected for further analysis based on antifungal, antibiofilm and cytotoxic results. Geraniol was tested using a dual-chamber co-culture model with TR146 cells infected with *C. albicans*, and THP-1 cells, used to mimic oral epithelium upon fungal infection. Expression of *Candida* enzymes (phospholipase–PLB and aspartyl proteases–SAP) and host inflammatory cytokines (interleukins: IL-1β, IL-6, IL-17, IL-18, IL-10, and Tumor necrosis factor–TNF) were analyzed. Lastly, geraniol *in vivo* toxicity was assessed using *Galleria mellonella*.

**Results:** MIC values obtained were 1.25–5 mM/mL for geraniol, 25-100 mM/mL for linalool, and 100–200 mM/mL for citronellal. Geraniol 5 and 50 mM/mL reduced yeast viability during biofilm analysis, only 500 mM/mL of linalool was effective against a 72 h biofilm and no biofilm activity was seen for citronellal. LD_50_ for TR146 and THP-1 were, respectively: geraniol 5.883 and 8.027 mM/mL; linalool 1.432 and 1.709 mM/mL; and citronellal 0.3006 and 0.1825 mM/mL. Geraniol was able to downregulate expression of fungal enzymes and host pro-inflammatory cytokines IL-1β, IL-6, and IL-18. Finally, safety *in vivo* parameters were observed up to 20 mM/Kg.

**Discussion:** Despite chemical similarities, geraniol presented better antifungal, antibiofilm activity, and lower cytotoxicity when compared to the other monoterpenes. It also showed low *in vivo* toxicity and capacity to downregulate the expression of fungal enzymes and host pro-inflammatory cytokines. Thus, it can be highlighted as a viable option for oral candidiasis treatment.

## 1 Introduction

Denture stomatitis is considered the most prevalent clinical form of oral candidiasis, constituting 70%–95% of the diagnosed cases, and is often associated with *Candida albicans* infection (Reinhardt et al., 2018; Vila et al., 2020). Pathogenesis of the condition can be approached as multifactorial with a fungal and inflammatory constituent. Host tendency to control pathogen proliferation, led by the immune system, is responsible for creating a characteristic local inflammatory pattern (D’Enfert et al., 2021). Conversely, inflammatory reactions caused by local trauma, such as ill-fitting dentures, may be associated with a favorable environment for *Candida* adhesion, proliferation, and tissue invasion (Reinhardt et al., 2018; D’Enfert et al., 2021).

Available antifungal drugs are somewhat scarcer than antibacterial drugs, and the increase in *Candida* resistance must not be underestimated. Additionally, those agents do not act in inflammatory host response (Costa-de-oliveira and Rodrigues, 2020). Therefore, identifying bioactive compounds that could act both in modulating the virulence factors of *C. albicans* and on host inflammatory response against the pathogen would likely improve treatment response.

The search for compounds derived from natural plants has gained attention over the years. However, a myriad of molecules has yet to be explored. Despite the rising concern about fungal resistance, no antifungals derived from natural compounds have been registered since 2006, which increases the need for new research in this field (Newman and Cragg, 2020). Geraniol, linalool, and citronellal are monoterpenes extracted from aromatic plants with the same molecular formula (C_10_H_18_O). Although the antimicrobial capacity of these compounds has already been discussed, neither the effect of these compounds on the inflammatory axis induced by *Candida* spp. nor the antibiofilm structure–activity relationship (SAR) has been well-investigated.

Here, we analyzed the antifungal activity, cytotoxicity, *in vivo* toxicity, distinctive antibiofilm SAR, and the influence of these compounds on the dysregulated inflammatory axis induced by *Candida* spp. Collectively, this study provides new insights into the mechanism of how monoterpenes modulate host function and opportunistic fungus infection.

## 2 Materials and methods

### 2.1 Monoterpenes

**FIGURE 1:**
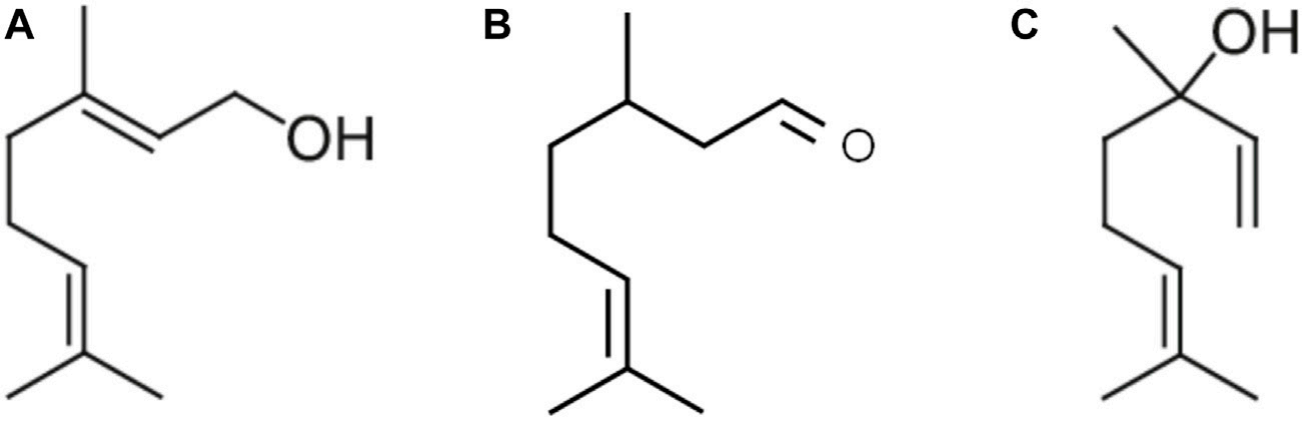
Acyclic monoterpene group: main components geraniol **(A)**, citronellal **(B)**, and linalool **(C)** contain two isoprene units and no cyclic portions in their structure.

The following compounds were used: geraniol (Alfa Aesar^®^, MA, United States), citronellal (MilliporeSigma^®^, MA, United States), and linalool (Alfa Aesar^®^, MA, United States) (Figure 1). Dimethyl sulfoxide 0.1% (DMSO, BDH Solvents^®^, GA, United States) was used as the vehicle.

### 2.2 Microorganisms

The following standard American Type Culture Collection (ATCC) reference yeast of *Candida* was used: *C. albicans* ATCC 321182, *C. albicans* ATCC 90028, *C. albicans* ATCC MYA 2876, *C. albicans* ATCC MYA 274, *Candida tropicalis* MYA 750, *Candida dublinienses* ATCC MYA 646, and *Candida glabrata* ATCC MYA 275.

### 2.3 Determination of minimal inhibitory concentration and minimal fungicidal concentration

Minimal inhibitory concentration (MIC) and minimal fungicidal concentration (MFC) of *Candida* strains were determined with the microdilution method as described by the CLSI document (Clinical and Laboratory Standards Institute, 2008). Thus, geraniol (40–0.31 mM/mL), citronellal, and linalool (800–12.5 mM/mL) were tested against all *Candida* species [2.5 × 10^3^ colony-forming unit (CFU)/mL]. A positive (fluconazole 0.4–0.0004 mM/mL), negative (medium only), and vehicle control (DMSO) were added to the test. Plates were incubated at 37°C–5% CO_2_ for 24 h and microbial growth was observed visually. Later, 10 μL of each well, with equal and/or higher concentrations of MIC were subcultured in Sabouraud dextrose agar medium (BD Difco^®^, NJ, USA) for 48 h, and visual growth was analyzed to determine the MFC. The ratio between MFC and MIC was used to determine compounds’ behavior as fungicidal (MFC/MIC < 4) or fungistatic (MFC/MIC ≥ 4) (Siddiqui et al., 2013).

### 2.4 Biofilm assay

Geraniol (5 and 50 mM/mL), citronellal (200 mM/mL), and linalool (50 and 500 mM/mL) were evaluated regarding their antibiofilm capacity, according to previous works (Seleem et al., 2016a; Chen et al., 2018).

To evaluate the initial biofilm inhibition, *C. albicans* ATCC^®^ MYA-2876 inoculum (1 × 10^6^ CFU/mL) was prepared using Yeast Nitrogen Base (YNB) medium (Sigma Aldrich^®^, MO, United States) supplemented with 50 mM of glucose (VWR Life Science^®^, PA, United States). Initial growth was then established for 24 h at 37°C and 5% CO_2_. Thereafter, the biofilms were treated daily with 10% v/v of the samples prepared in 1% DMSO, until it reached 72 h. At each 24 h time, the supernatant was removed, and the biofilm was washed twice with phosphate buffer solution (PBS) (Lonza Bioscience^®^, MD, United States); a measure of 900 μL of fresh YNB medium with 100 µL of the test compounds were added to the wells. The vehicle control was 1% DMSO and the positive control was fluconazole 0.01 mM/mL (10 × MIC). A mature biofilm was formed following the same concept described above. However, the biofilm remained untouched for 72 h. Treatments were also applied as described.

After the treatment time of both methods, adhered biofilms were collected by scraping the bottom of each well plate and suspending in PBS, which was then centrifuged at 10,000 rpm for 5 min. The biomass (dry weight) of each biofilm sample was obtained by discarding the supernatant and placing the samples in a speed vacuum to dry for 40 min. CFU was determined by counting the colonies at Sabouraud dextrose agar plates, which were incubated at 37°C—5% CO_2_. Data were normalized based on the CFU/mL/dry weight of the biofilm sample.

### 2.5 Cytotoxicity assay

Cytotoxic effect of geraniol (500–0.05 mM/mL), citronellal (50–0.005 mM/mL) and linalool (500–0.05 mM/mL) on THP-1 (ATCC TIB-202) human monocytes cells and oral squamous cell carcinoma cell line TR146 (ECACC 10032305) were assessed with resazurin fluorometric method (Cell Titer Blue Viability Assay, Promega Corp^®^, WI, USA).

THP-1 and TR146 cells (2.5 × 10^5^ cells/mL) were cultured, respectively, in RPMI (Roswell Park Memorial Institute) and Ham’s F12 medium with L-glutamine (Lonza Bioscience^®^, MD, United States), mediums were supplemented with 10% of fetal bovine serum (FBS, Gibco, Invitrogen, MA, United States) and penicillin/streptomycin (Lonza, MD, United states). Cells were cultured in 24-well plates followed by compound addition (10% v/v). After 24 h, cell titer blue was added to each well and the plates were incubated for 3 h. The supernatant fluorescence was read in a microplate reader with excitation of 555 nm, emission of 585, and 570 nm cutoff (O’Brien et al., 2000).

### 2.6 Dual-chamber *in vitro* model

**FIGURE 2:**
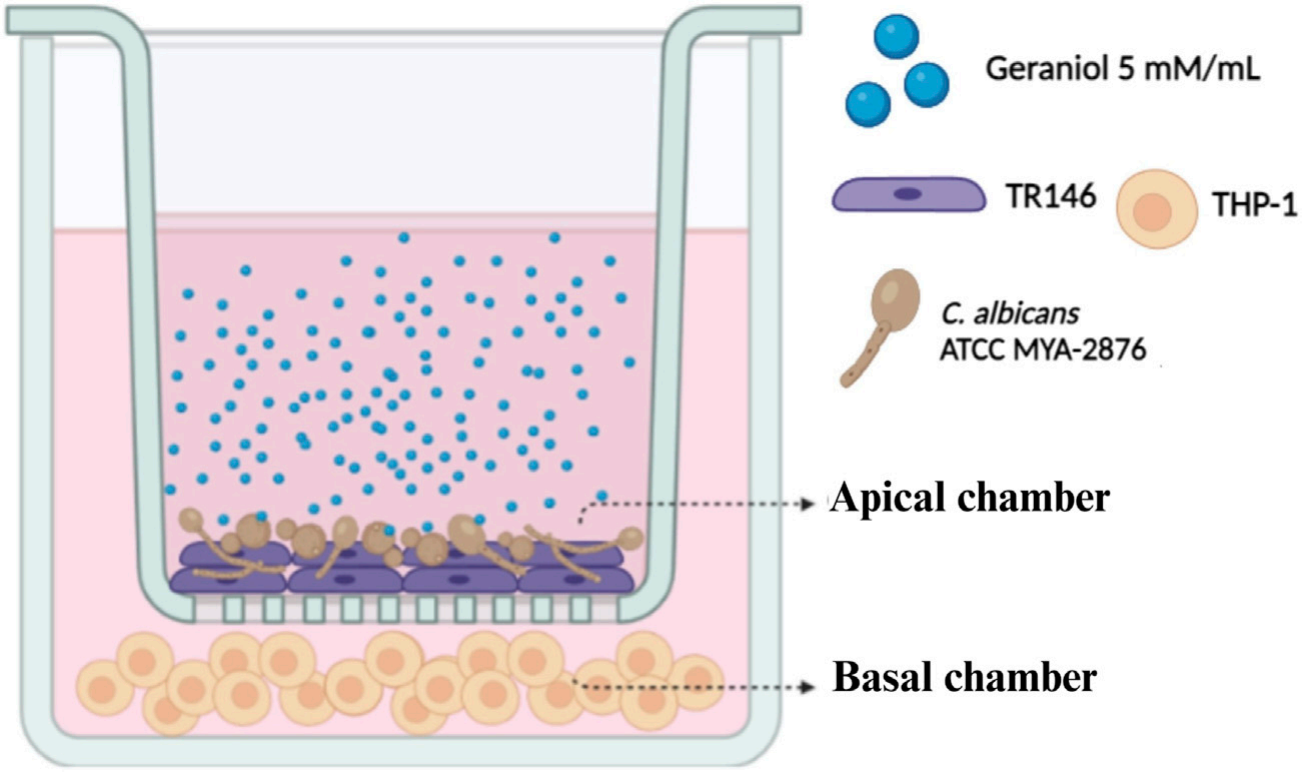
Dual-chamber *in vitro* model. The apical chamber represents the first barrier of the epithelial layer, in which TR146 cells were subcultured and subsequently infected with *C. albicans* American Type Culture Collection (ATCC) MYA 2876. THP-1 cells were placed in the basal chamber to evaluate the influence of geraniol (5 mM/mL) on the dysregulated inflammatory axis induced by *Candida* spp.

Based on antifungal, antibiofilm, and cytotoxic results, geraniol was selected for further analysis. Dual-chamber *in vitro* system (Pasetto et al., 2014) (Figure 2) was used to mimic oral epithelium upon fungal infection. TR146 cells (2 × 10^6^ cells/mL) were seeded, using Ham`s F12 with L-glutamine, and 10% FBS, in cell culture inserts with a PET membrane of 1 µm pore size, and 452.4 mm^2^ of culture surface (Greiner Bio-One^®^, NC, United States). Inserts were placed in a 6-well plate and incubated. The trans epithelial electric resistance (TEER) of each insert well was measured daily to assess the confluence of the cells using a Millicell-ERS Volt-Ohm Meter (Millipore, MA, United States) until the optimal TEER (30 Ω/cm^2^) was reached on day 6. Afterward, inserts were transferred to a new plate containing THP-1 cells (2 × 10^5^ cells/mL) in RPMI medium. *Candida albicans* inoculum (1 × 10^5^ CFU/mL), prepared in RPMI without FBS, was then transferred to the apical chamber. Lastly, geraniol treatment (5 mM/mL—MIC) was added (10% v/v), and the plate was incubated for 4 h. DMSO and medium only were used as control.

### 2.7 RNA extraction and quantitative real-time RT-PCR

Succeeding the 4 h of treatment with geraniol, RNA was isolated from THP-1 cells and *C. albicans* using respectively Ilustra™ RNAspin Mini (GE Healthcare, IL, United States) and RiboPure™ Yeast (Invitrogen, VLN, Lithuania). Real-time reverse transcription polymerase chain reaction (RT-PCR) was conducted in a thermocycler (QuantStudio 3 RT-PCR System, Thermo Fisher Scientific, Rockford, IL, United States) using QuantiNova^®^ SYBR^®^ Green RT-PCR Kit (QIAGEN^®^, Hilden, Germany) and the primers described in Table 1. Manufacture instructions were followed during the experiment. All data were normalized using housekeeping genes, and relative gene expression was achieved with ^ΔΔ^Ct method (Nailis et al., 2010; Seleem et al., 2016a; Seleem et al., 2016b; Chen et al., 2018).

**TABLE 1:** Primers used for host and fungal gene expression using reverse transcription polymerase chain reaction (RT-PCR).

Primers used for RT-PCR analysis	
Cytokine gene expression (QuantTect® Primer Assay—QIAGEN®)	IL-1β
	IL-6
	IL-17
	IL-18
	IL-10
	TNF
	GAPDH (Housekeeping)
*C. albicans* primers (Nails et al., 2010)	Secreted aspartyl proteinases-1 (SAP-1)
	Phospholipase (PLB-1)
	ACT-1 (Housekeeping)

### 2.8 Co-culture model for fluorescence microscopy

TR146 cells were cultured as described above in a 24-well plate. The medium was then replaced with *C. albicans* inoculum (5 × 10^4^ CFU/mL) prepared in Ham’s F12 with L-glutamine mixed with geraniol treatment (5 mM/mL—MIC), and the plate was incubated for 24 h. DMSO and fluconazole (0.01 mM/mL) were added as test controls. TR146 cell viability was observed using LIVE/DEAD™ Viability/Cytotoxicity Kit (Invitrogen, MA, USA), and *C. albicans* was stained with calcofluor white (Sigma Aldrich, San Luis, MO, USA). Fluorescent images of the double staining were captured using fluorescence microscopy (Keyence All-in-One BZ-X810 Fluorescence Microscope, Itasca, IL, USA).

### 2.9 *In vivo* acute toxicity of geraniol in the *G. mellonella* larvae model

Different doses of geraniol (0.8–8,000 mM/kg) were injected into the left proleg of 10 randomly selected healthy-looking larvae using a Hamilton Syringe (Hamilton, Reno, NV, USA). A vehicle control group (DMSO) and an injection-only group served as test controls. Larvae were incubated at 30°C, and their survival was evaluated until the maximum time of 96 h (Loh et al., 2013; Rochelle et al., 2016; Champion et al., 2018).

### 2.10 Statistical analysis

All *in vitro* analyses were realized in triplicates at three distinct times. Data were analyzed using GraphPad Prism software (version 8.02). When applicable, the results were expressed as mean and standard deviation. Data were analyzed statistically using one-way analysis of variance and Dunnett’s multiple comparison tests in relation to the negative or vehicle control. Lastly, LD_50_ for cytotoxic tests was assessed by non-linear regression. Significance was accepted for a value of *p* ≤ 0.05.

## 3 Results

### 3.1 Determination of minimal inhibitory concentration and minimal fungicidal concentration

Geraniol (MIC 1.25–5 mM/mL, MFC 10–20 mM/mL) presented antifungal activity against all tested strains with lower MIC and MFC values when compared with linalool (MIC 25–100 mM/mL, MFC 25–100 mM/mL) and citronellal (MIC 100–200 mM/mL, MFC 200 mM/mL) (Table 2).

**TABLE 2:** Minimal inhibitory concentration (MIC) and minimal fungicidal concentration (MFC) of geraniol, linalool, citronellal, and fluconazole according to the species of *Candida*. The ratio obtained from MFC/MIC is also shown.

Microorganism	Geraniol			Linalool			Citronellal			Fluconazole			
	MIC mM/mL	MFC mM/mL	MIC/MFC	MIC mM/mL	MFC mM/mL	MIC/MFC	MIC mM/mL	MFC mM/mL	MIC/MFC	MIC mM/mL		MFC mM/mL	MIC/MFC
*C. albicans* ATCC 321182	1.25	10	>4	25	50	<4	100	200	<4	0.1	0.4		>4
*C. albicans* ATCC MYA 274	2.5	10	4	100	100	<4	100	200	<4	0.0008	0.1		>4
*C. albicans* ATCC MYA 2876	5	20	4	50	100	<4	200	200	<4	0.001	0.1		>4
*C. albicans* ATCC MYA 90028	1.5	10	>4	50	50	<4	200	200	<4	0.0008	0.2		>4
*C. dublinienses* ATCC MYA 646	2.5	10	4	100	100	<4	200	200	<4	0.0008	0.1		>4
*C. tropicalis* ATCC 750	1.5	20	4	100	100	<4	200	200	<4	0.001	0.4		>4
*C. glabrata* ATCC MYA 275	5	20	4	100	100	<4	200	200	<4	0.0008	0.4		>4

Fungicidal (MFC/MIC < 4) and fungistatic (MFC/MIC ≥ 4; Siddiqui et al., 2013).

### 3.2 Antibiofilm activity

**FIGURE 3:**
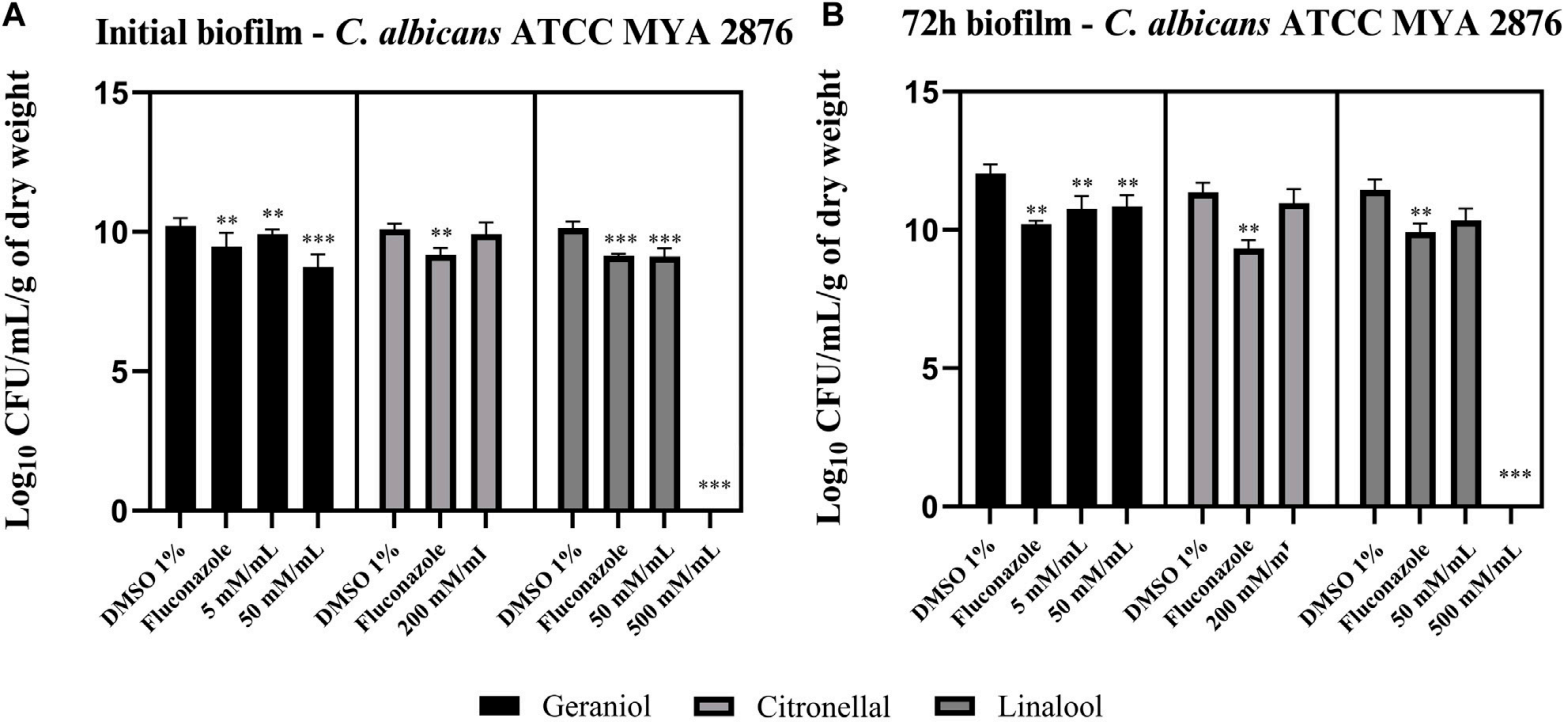
Monoterpene effect upon an initial biofilm **(A)** and a 72-h biofilm **(B)** of *C. albicans* ATCC MYA 2876. Geraniol 5 mM/mL– minimal inhibitory concentration (MIC) and 50 mM/mL—10 × MIC were able to reduce fungal viability in both the initial and 72-h biofilm. Linalool 50 mM/mL—MIC and 500 mM/mL—10 × MIC inhibited the initial biofilm formation, whereas only 10 × MIC concentration was able to reduce the 72-h biofilm viability. Lastly, citronellal 200 mM/mL—MIC could not reduce biofilm viability for both biofilm models. Comparisons were made with the vehicle control [dimethyl sulfoxide (DMSO) 1%]. Results are expressed as CFU/mL/g of dry weight count, and significance values were considered *p ≤ 0.05, **p ≤ 0.01, ***p ≤ 0.001, and ****p ≤ 0.0001.

Geraniol 5 and 50 mM/mL showed a significant (*p* < 0.05) reduction in *C. albicans* biofilm viability–ATCC MYA 2876 (Figure 3A and B). Linalool was able to reduce CFU/mL/g of the dry weight of the initial biofilm at both tested concentrations (Figure 3A). However, only 50 mM/mL had a significant effect on the 72-h biofilm (Figure 3B). Conversely, citronellal (200 mM/mL–MIC) did not show any biofilm activity when compared with the control (*p* > 0.05) (Figure 3A and B).

### 3.3 Cytotoxicity assay

**FIGURE 4:**
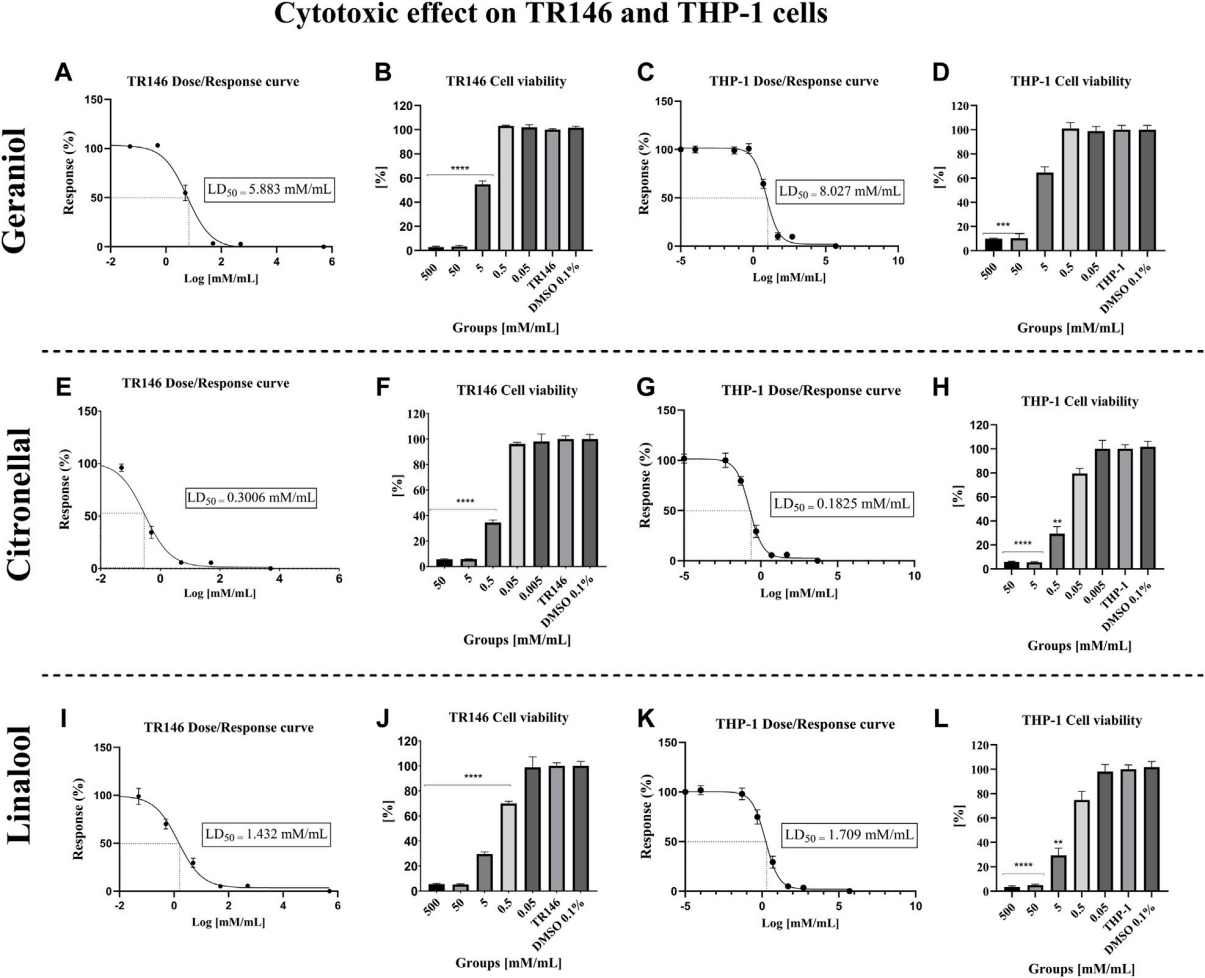
Cytotoxic effect of the monoterpenes on TR146 and THP-1 cells after 24 h of treatment. The LD_50_ values obtained for TR146 and THP-1 cells, respectively were: 5.883 mM/mL **(A)** and 8.027 mM/mL **(C)** for geraniol; 0.3006 mM/mL **(E)** and 0.1825 mM/mL **(G)** for citronellal; and 1.432 mM/mL **(I)** and 1.709 mM/mL **(K)** for linalool. Percentages of TR146 cell viability was significantly different from the vehicle control from 5 mM/mL onward for geraniol **(B)**, and from 0.5 mM/mL onward for citronellal **(F)** and linalool **(J)**. Regarding THP-1 cells, significant differences in cell viability was seen from 50 mM/mL onward for geraniol **(D)**, 0.5 mM/mL for citronellal **(H)** and 5 mM/mL for linalool **(L)**. TR146 and THP-1: Cells only; DMSO 0.1%: Vehicle control. significance values were considered as *p ≤ 0.05, **p ≤ 0.01, ***p ≤ 0.001, and ****p ≤ 0.0001.

**FIGURE 5:**
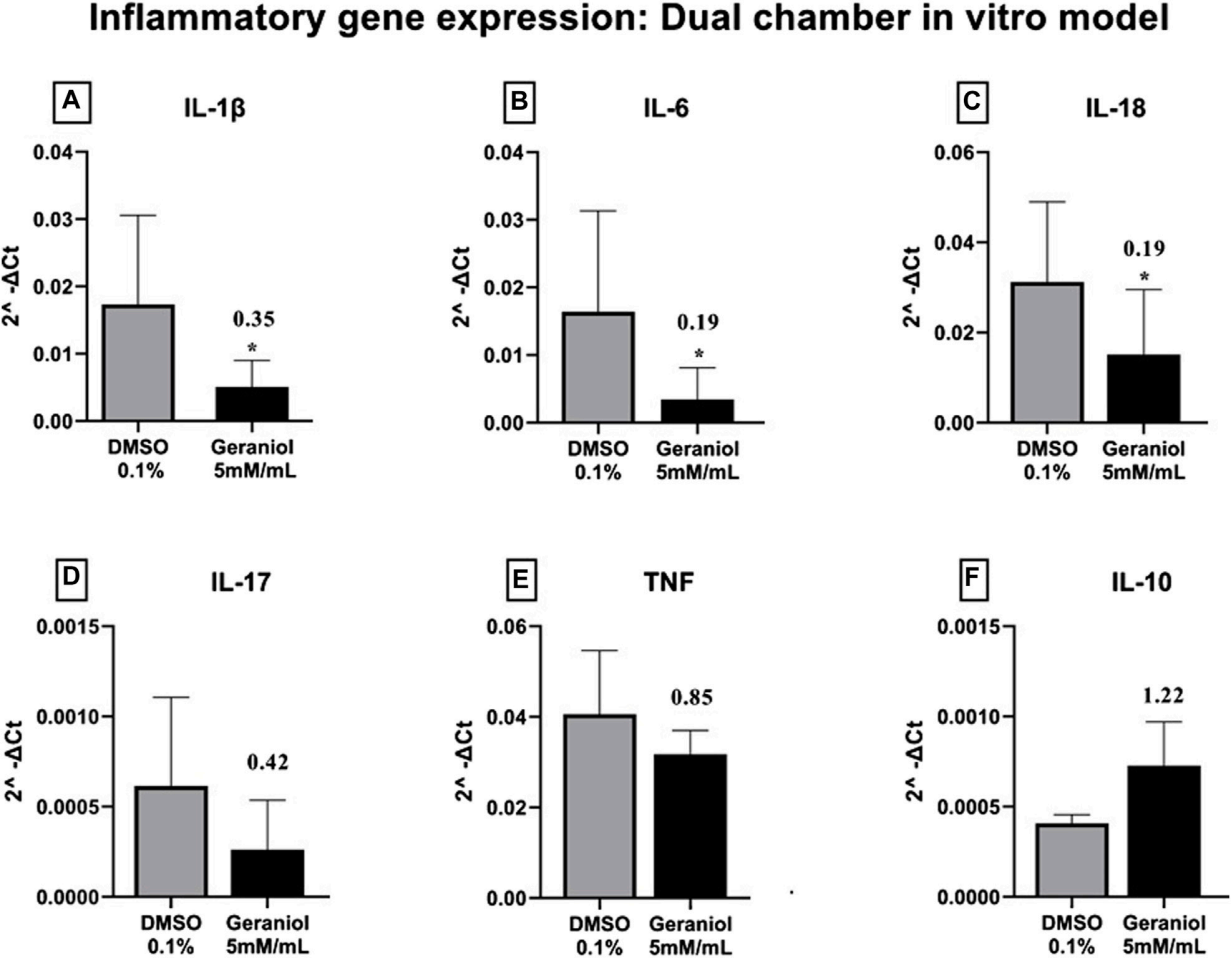
Relative gene expression of **(A)** IL-1β, **(B)** IL-6, **(C)** IL-18, **(D)** IL-17, **(E)** TNF, and **(F)** IL-10 of THP-1 cells after 4 h of *C. albicans* MYA 2876 infection in a dual-chamber *in vitro* model and treatment with geraniol 5 mM/mL. The fold change was established as relative to the vehicle control group DMSO 0.1%. Significance values were considered as *p ≤ 0.05.

Geraniol’s LD_50_ values for TR146 and THP-1 cells were 5.883 mM/mL (Figure 4A) and 8.027 mM/mL (Figure 4C), respectively. Citronellal’s LD_50_ was 0.3006 mM/mL for TR146 cells (Figure 4E) and 0.1825 mM/mL for THP-1 cells (Figure 4G). Lastly, linalool’s LD_50_ values were 1.432 mM/mL for TR146 cells (Figure 4I) and 1.709 mM/mL for THP-1 cells (Figure 4K). Additionally, the percentage of TR146 cell viability was significantly different from the vehicle control (*p* < 0.05) from 5 mM/mL onward for geraniol (Figure 4B) and from 0.5 mM/mL onward for citronellal (Figure 4F) and linalool (Figure 4J). Regarding THP-1 cells, geraniol showed significant differences in cell viability from 50 mM/mL onward (Figure 4D), whereas this value was 0.5 mM/mL for citronellal (Figure 4H) and 5 mM/mL for linalool (Figure 4L).

### 3.4 Inflammatory cytokine gene expression

The gene expression of pro-inflammatory genes IL-1β (Figure 5A), IL-6 (Figure 5B), and IL-18 (Figure 5C) were significantly (*p ≤* 0.05) downregulated after geraniol treatment. Lastly, IL-17 (Figure 5D) and tumor necrosis factor (TNF) (Figure 5E) were downregulated, and IL-10 (Figure 5F) was upregulated but with no statistical difference (*p >* 0.05) to the control group.

### 3.5 Proteolytic enzyme gene expression

**FIGURE 6:**
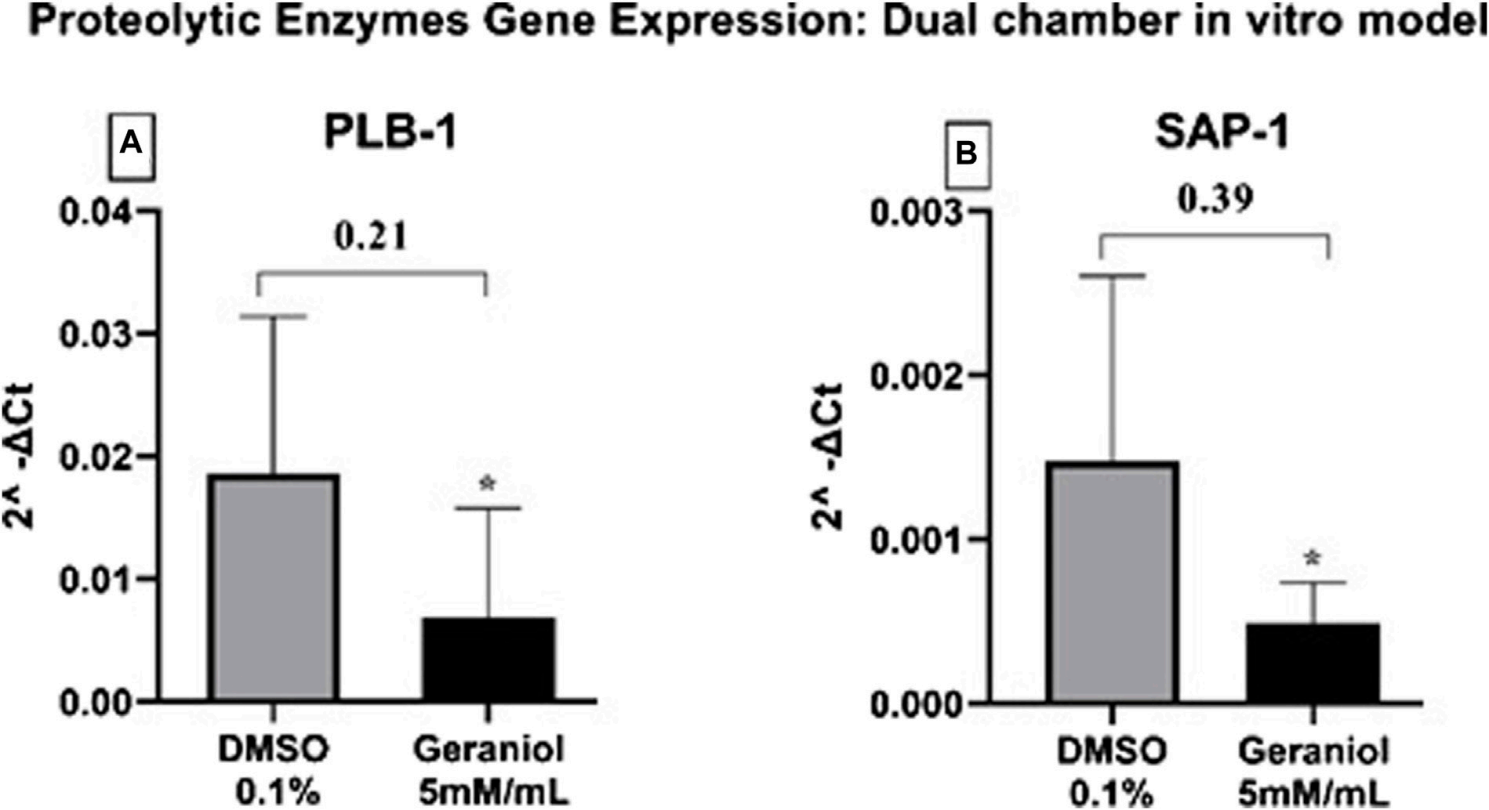
Relative gene expression of **(A)** PLB-1 and **(B)** SAP-1 secreted by *C. albicans* MYA 2876 after 4 h of infection in a dual-chamber *in vitro* model and treatment with geraniol 5 mM/mL. The fold change was established as relative to the vehicle control group DMSO 0.1%. Significance values were considered as *p ≤ 0.05.

Geraniol was able to significantly (*p ≤* 0.05) downregulate the expression of SAP-1 and PLB-1 genes (Figure 6).

### 3.6 Co-culture model for fluorescence microscopy

**FIGURE 7:**
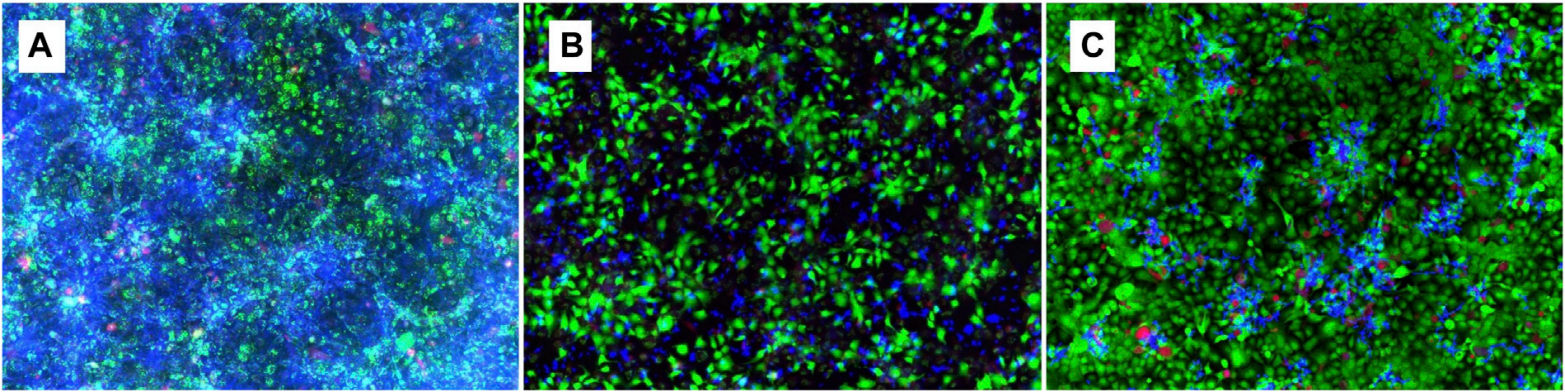
Fluorescence microscopy of 24 h geraniol 5 mM/mL treatment **(B)** in a co-culture of TR146 cells and *C. albicans*. DMSO 0.1% was used as control **(A)** and fluconazole 0.01 mM/mL as positive control **(C)**. Magnification power of 20×.

Geraniol showed a decrease in *Candida* growth distribution (Figure 7B), as indicated by a reduction in fluorescent blue color and less dense accumulation of cell clusters in comparison to the vehicle control (Figure 7A). Additionally, a restricted hyphal presence was noticed when compared to both vehicle (Figure 7A) and positive control (Figure 7C).

### 3.7 *In vivo* toxicity of geraniol in the *G. mellonella* larva model

**FIGURE 8:**
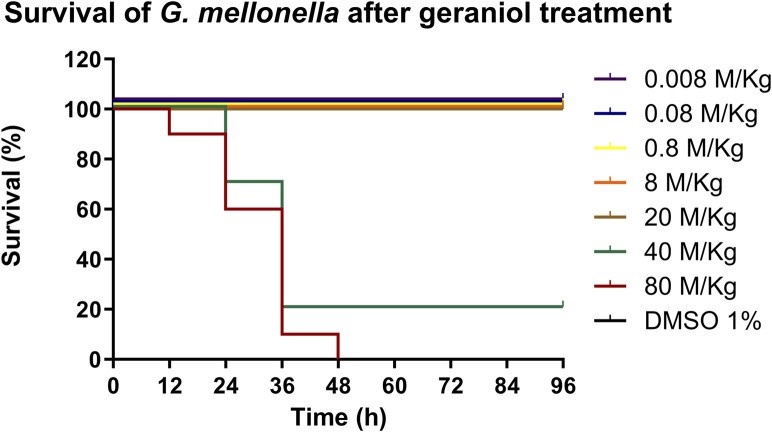
*In vivo* toxicity of geraniol (0.008–80 M/kg) in the *G. mellonella* larva model. Geraniol did not interfere with larval survival up to 20 M/kg. DMSO 1%: vehicle control; Control: injection only.

No sign of toxicity was seen in the larvae under geraniol treatment up to 20 M/kg when compared with the control (*p* > 0.05) (Figure 8).

## 4 Discussion

*Candida* species resistance to traditional antifungal agents, including triazoles, presents a significant obstacle, particularly in immunocompromised individuals, such as those with human immunodeficiency virus. Over the past four decades, the Food and Drug Administration has granted approval for a mere 34 new antifungal agents, 20% of these novel molecules derivers from natural products, which underscores their noteworthy potential in combating fungal infections (Newman and Cragg, 2020). Even though geraniol, citronellal, and linalool have the same molecular formula (C_10_H_18_O), differences in the antifungal, antibiofilm, and cytotoxic effects could be seen in the present study. Based on SAR correlation, a chemical structure difference directly relates to changing compounds’ biological properties. However, few studies have analyzed the SAR correlation regarding monoterpenes’ biological activities, and this relation to antifungal or antimicrobial activities has not yet been well-established.

The overall three-dimensional structure of these molecules, influenced by the arrangement of isoprene units and the hydroxyl group, can affect their interactions with biological targets and exert influence upon components’ effectiveness. However, specific mechanisms remain poorly characterized (Christianson, 2017; Singulani et al., 2018; Badawy et al., 2019; Mahizan et al., 2019). Differences were initially seen in MIC results, in which geraniol presented lower values compared with linalool and citronellal. This has also been seen by Singulani et al. (2018); the authors found that geraniol was more effective against *C. albicans* strains than linalool. Previous studies have found MIC values for geraniol, such as 225 μg/mL (equivalent to 1.45 mM/mL) for *C. albicans* and 300 μg/mL (equivalent to 1.94 mM/mL) for non-*albicans Candida* species (Singh et al., 2016). Additionally, opposing Singh et al. (2019), in which geraniol showed a fungicidal effect on *C. albicans* strains, we found a fungistatic profile for all *Candida* strains tested (Siddiqui et al., 2013). Conversely, a fungicidal pattern was seen for citronellal and linalool. The fungistatic profile of a compound might constitute a desirable effect rather than the complete elimination of the pathogen. *Candida* spp. is an important component of the oral microbiome, present in immunocompetent individuals as a commensal pathogen. Thus, controlling its virulence factors should prevent the rise of pathogenic strains and maintain microbiome homeostasis (Bhattacharya et al., 2020; Lemberg et al., 2022).

Regarding antibiofilm activity, MIC concentration of geraniol 5 mM/mL was effective in reducing *C. albicans* biofilm viability. In contrast, linalool was only effective against biofilm formation at a 10 × MIC concentration, and citronellal had no antibiofilm activity up to 200 mM/mL. The ability of *C. albicans* to form biofilm is one of the major virulence factors related to candidiasis pathogenesis, primarily because of the extracellular polymeric matrix that enfolds the layers of microorganisms. The biofilm structure provides nutrients and protection against several factors, such as aggression from toxins, pH changes, host immune response, and diffusion of antifungal agents. (Vila et al., 2020; D’Enfert et al., 2021). Despite the stable environment created in the biofilm structure, geraniol was effective at MIC concentration. Diverging from Kaypetch et al.’s (2022) study, the authors found that concentrations of 640 μg/mL, equivalent to 2.5-fold MIC, were ineffective against *Candida* biofilm formation, positive effects were only seen at 5 and 10 × MIC.

The literature reports a possible correlation between monoterpenes’ mechanism of action and the induction of membrane disruption of microorganisms. The ergosterol-binding capacity of the compounds results in channel formation and increases fluidity and permeability, leading to the destabilization of fungal cell membranes. Such activity may be associated with its non-polar character, which disrupts fungal lipid structure. Additionally, alcohol moieties present in monoterpenes, such as geraniol, may also suggest antifungal activity, as well as the presence of hydroxyl groups, oxygen functions, and delocalized electrons, which are among the antimicrobial determining factors (Singh et al., 2016; Mahizan et al., 2019; Lira et al., 2020). Studies have also shown that geraniol is capable of altering ATPase activity in the plasma membrane, causing mitochondrial dysfunction, and reducing hyphal formation (Singh et al., 2016; Badawy et al., 2019; Lira et al., 2020; Kaypetch et al., 2022).

Differences among the compounds’ activity were also seen in the cytotoxic response in which geraniol also demonstrated better results, with a lower cytotoxic profile when compared with citronellal and linalool. The cytotoxic assay with TR146 and THP-1 cells was an essential step of the present study, acting as a parameter to determine the compound therapeutic concentration used in the dual-chamber co-culture model. Geraniol had an LD_50_ of 5.883 mM/mL and 8.027 mM/mL, respectively, for TR146 and THP-1 cells, which indicates a minimal interference in cell viability during further tests when using MIC concentration (5 mM/mL).

Based on antifungal, antibiofilm, and cytotoxic results, geraniol was selected for the dual-chamber co-culture model to assess the influence of this compound on the dysregulated inflammatory axis induced by *Candida* spp. Studies have shown that the innate immune response to *C. albicans* is related to the expression of cytokines such as IL-6, IL-8, IL-17, and TNF. Modulating the overexpression of inflammatory cytokines is relevant to inflammatory disease pathogenesis, tissue degradation, and carcinogenesis (Murata, 2018; Gupta et al., 2021; Ho et al., 2021).

Isoprene units of monoterpenes can be related to SAR modulation of anti-inflammatory activity by influencing interactions with biological membranes and certain proteins, which affects cell membrane penetration, bioavailability, and, subsequently, their anti-inflammatory activity. Additionally, hydrogen bonding may be involved in binding to specific receptors or enzymes, influencing the anti-inflammatory activity. Herein, we could see a significant (*p <* 0.05) downregulation of IL-1β, IL-6, and IL-18 after geraniol treatment. Induction of IL-1β and IL-6 during oral candidiasis infection seems to be related to hyphal formation, indicating an interesting host mechanism of detecting yeast switch from commensal to pathogenic (Nishikawa et al., 2023).

Based on the discussed parameter, we can assume that IL-1β and IL-6 downregulation may be associated with hyphal depletion seen in fluorescent microscopy after geraniol treatment (Figure 7B), an important virulence factor of *C. albicans* (D’Enfert et al., 2021)*.* Additionally, geraniol treatment was able to downregulate the PLB-1 and SAP-1 gene expression. Those enzymes have a critical role in fungal pathogeneses, such as yeast-hyphal transformation, adhesion, and tissue invasion (Kumar et al., 2017; D’Enfert et al., 2021; Kulshrestha and Gupta, 2023).

Even though we could not see a statistical difference, a downregulation pattern was seen for TNF and IL-17, important proinflammatory cytokines involved in host response in *C. albicans* infection (Ramírez-Amador et al., 2017; Rai et al., 2022). Conversely, an upregulation pattern was seen for the anti-inflammatory interleukin IL-10, an important component of the reestablishment of immune homeostasis (Rutz and Ouyang, 2016; Ouyang et al., 2021). Further molecular studies should be conducted to confirm its modulation. Geraniol immunomodulatory action upon cytokines, such as IL-1β, IL-6, TNF-α, IFN-γ, and IL-10, has already been discussed. However, to the best of our knowledge, no other study evaluated geraniol inflammatory modulation under *Candida* infection (Wu et al., 2020; El Azab et al., 2022; Ammar, 2023).

Furthermore, the *G. mellonella* test showed a non-toxic profile for geraniol up to 20 M/kg. The innate immune response of *G. mellonella* shares several properties with the mammalian immune system, also it is more advanced than other invertebrates’ models, such as nematodes. Thus, it qualifies as a well-accepted scientific method to be used in a preclinical stage (Champion et al., 2018). Safety parameters were also assessed in other *in vivo* studies, but with lower concentrations and with a less complex model (Singh et al., 2019). Additionally, no present safety concern regarding geraniol has been discriminated against, based on estimated intake levels, by the Joint FAO/WHO Expert Committee on Food Additives (World Health Organization, 2004). However, more robust *in vivo* tests are required to validate our findings.

Based on the present results, the overall three-dimensional structure of these molecules may affect their interactions with biological targets. Thus, further studies should be conducted to fully understand the influence of those three monoterpenes’ chemical structure and the difference in the effectiveness of the biological activities. Additionally, we can highlight geraniol as a viable option for oral candidiasis treatment considering the low *in vivo* toxicity, antifungal activity, and anti-inflammatory response. Therefore, the present results can sustain more studies to assess its efficacy and safety in a more clinically robust setting.

## 5 Conclusion

Our findings highlight the promising aspects of geraniol over citronellal and linalool, as well as emphasize the SAR correlation of those monoterpenes. Geraniol demonstrated better antifungal and antibiofilm activities, with lower cytotoxicity and *in vivo* toxicity. Additionally, it was able to interfere with downregulating *Candida* spp.-induced inflammatory axis and minimized *Candida* proteolytic enzyme expression.

## Data Availability

The original contributions presented in the study are included in the article/Supplementary Material; further inquiries can be directed to the corresponding authors.
